# A Simulation-Based Stress-Testing Framework for Evaluating the Transportability of Imaging-Derived Logistic Risk Models Across Cutaneous Lesion Phenotypes

**DOI:** 10.3390/diagnostics16131961

**Published:** 2026-06-24

**Authors:** Betül Tiryaki Baştuğ, Özlem Türelik, Sinan Topuz, Buket Dursun Çoban, Hatice Gencer Başol

**Affiliations:** 1 Department of Radiology, Faculty of Medicine, Bilecik Şeyh Edebali University, Bilecik 11000, Türkiye; 2 Department of Pathology, Faculty of Medicine, Bilecik Şeyh Edebali University, Bilecik 11230, Türkiye; ozlem.turelik@bilecik.edu.tr; 3 Department of Plastic Surgery, Bilecik Training and Research Hospital, Bilecik 11230, Türkiye; sinantopuz29@gmail.com (S.T.); buketdursun@gmail.com (B.D.Ç.); 4 Department of Dermatology, Bilecik Training and Research Hospital, Bilecik 11230, Türkiye; haticegencer17@hotmail.com

**Keywords:** model transportability, structural robustness, simulation-based methodology, structural perturbation, logistic regression, calibration, decision curve analysis, imaging-based risk stratification

## Abstract

**Background**: Imaging-based logistic models are widely used for non-invasive risk stratification; however, their structural robustness and transportability across heterogeneous biological contexts remain insufficiently examined. **Purpose**: This study aimed to develop a simulation-based stress-testing framework to evaluate the structural robustness and transportability of a radiology-adapted logistic risk model across distinct cutaneous lesion phenotypes under both aligned and structurally perturbed conditions. **Methods**: A simulation-based methodological framework was implemented using three synthetic cohorts representing nodular, subcutaneous, and vascular lesion phenotypes (*n* = 2000 per cohort). Model performance was evaluated under naïve transfer, recalibration, and revision conditions. To address potential structural alignment bias, additional simulation scenarios incorporating coefficient perturbations, nonlinear transformations, and interaction effects were used to generate outcome processes partially independent from the original model structure. Model performance was assessed using discrimination (ROC-AUC, PR-AUC), calibration metrics, decision curve analysis, and Monte Carlo-based stability assessments. **Results**: Under naïve transfer, discrimination remained stable across phenotypes (ROC-AUC ≈ 0.78–0.84). Calibration shifts were observed but were effectively corrected through recalibration. Under structurally perturbed outcome generation, discrimination showed only modest reduction, while overall performance patterns remained consistent. Structural variables demonstrated high transferability, whereas vascular features exhibited phenotype-dependent variability. Decision curve analysis indicated consistent clinical utility across relevant thresholds. **Conclusions**: The radiology-adapted logistic model demonstrated structural robustness across heterogeneous phenotype conditions, with performance variations driven primarily by calibration differences rather than structural failure. Importantly, robustness was preserved under conditions of structural perturbation, supporting the model’s stability beyond idealized alignment assumptions. These findings suggest that simulation-based stress-testing frameworks provide a rigorous methodological approach for evaluating model transportability prior to large-scale clinical validation.

## 1. Introduction

Non-invasive diagnostic strategies are increasingly shaping modern medicine, particularly in fields where early differentiation between benign and malignant lesions directly influences clinical outcomes. In dermatologic oncology, pigmented superficial skin lesions represent a wide diagnostic spectrum, ranging from completely benign nevi to malignant tumors with invasive potential. While histopathology remains the reference standard, reliance on biopsy-based confirmation presents inherent limitations, including procedural morbidity, cosmetic concerns, patient anxiety, and healthcare resource burden. These challenges have fueled growing interest in imaging-supported, risk-stratified diagnostic pathways that can guide pre-biopsy decision-making [1,2].

High-frequency ultrasonography, especially when combined with Doppler assessment, has emerged as a valuable modality for evaluating superficial cutaneous lesions. Grayscale imaging provides structural information such as lesion size, depth, and border characteristics, while Doppler ultrasound reveals vascular architecture and hemodynamic behavior associated with tumor angiogenesis. Together, these features offer a non-invasive window into both morphological and biological characteristics of skin lesions. However, despite accumulating evidence supporting individual sonographic markers, most previous studies have remained descriptive, parameter-specific, or limited to narrow lesion categories. Few investigations have attempted to integrate structural and vascular imaging features into unified predictive frameworks capable of generating quantitative malignancy risk estimates [3,4].

Recently, a radiology-adapted logistic regression model was proposed to address this gap by systematically combining grayscale and Doppler-derived parameters for non-invasive risk stratification of pigmented superficial skin lesions. That model demonstrated encouraging diagnostic performance, suggesting that morphologic and hemodynamic ultrasound features could be translated into clinically meaningful risk scores. Nevertheless, an important methodological question remains unanswered: are such imaging-based logistic risk models intrinsically tied to the specific lesion population in which they were developed, or can their structural logic be generalized to other cutaneous lesion phenotypes?

This issue relates to the concept of model transportability, a critical but underexplored topic in imaging-based prediction research. Predictive models often show strong performance in development cohorts but degrade when applied to populations with different biological behavior, lesion morphology, or imaging characteristics. In cutaneous imaging, lesion phenotypes differ substantially between pigmented superficial lesions, nodular dermal tumors, subcutaneous masses, and vascular lesions. These phenotypic variations may alter the distribution and diagnostic weight of structural and vascular parameters, potentially affecting model calibration, discrimination, and clinical utility [5,6].

Understanding which components of an imaging-based logistic model are phenotype-independent (transferable) and which are lesion-type-specific (non-transferable) is essential for advancing non-invasive diagnostic modeling from local pilot tools toward broadly applicable clinical decision-support systems. However, direct evaluation of model generalizability across multiple lesion populations typically requires large, heterogeneous, multi-institutional datasets that are rarely available at early methodological stages [7,8].

To address these methodological gaps, simulation-based modeling offers a controlled framework in which model structure can be evaluated independently from population-specific data distributions. By generating phenotype-specific synthetic cohorts, it becomes possible to investigate whether predictive relationships between imaging features and risk remain stable under varying biological contexts.

However, an important methodological consideration in simulation-based modeling is the potential structural alignment between the data-generating process and the evaluated model. When outcome variables are generated using assumptions closely related to the analytical framework, there is a risk that model performance may be partially influenced by this alignment rather than reflecting true robustness. Therefore, careful interpretation of simulation-based findings requires additional evaluation under conditions of structural perturbation or misspecification. Incorporating such approaches may provide a more rigorous assessment of whether observed model behavior reflects genuine transportability or methodological artifact. In the present study, this consideration was addressed by incorporating additional simulation scenarios designed to evaluate model behavior under conditions of structural perturbation and partial model misspecification.

In this context, in silico simulation modeling provides a scientifically robust and ethically neutral framework for investigating model behavior under controlled variations in lesion phenotype, parameter distributions, and disease prevalence. By generating synthetic cohorts that reflect plausible structural and vascular feature patterns across different cutaneous lesion groups, simulation studies allow researchers to isolate the intrinsic properties of a model’s mathematical structure, independent of specific patient datasets [9,10].

In recent years, predictive modeling approaches have increasingly been incorporated into medical imaging research to support non-invasive risk stratification. Logistic regression models remain widely used because they provide interpretable relationships between imaging-derived variables and clinical outcomes while maintaining statistical transparency. However, an important methodological challenge is model transportability—that is, whether a model developed in one population maintains validity when applied to different biological contexts or imaging environments. In dermatologic imaging, variations in lesion morphology, vascular architecture, and growth patterns across different lesion phenotypes may substantially alter predictor distributions and influence model calibration and discrimination [5,6].

Therefore, the present study was designed as a methodological in silico investigation to evaluate the generalizability of a radiology-adapted logistic risk model across distinct cutaneous lesion phenotypes. Specifically, we aimed to:

- Assess the performance of the original model structure when applied to simulated cohorts representing nodular, subcutaneous, and vascular lesion phenotypes.
- Identify which imaging-derived variables demonstrate stable predictive behavior across lesion types (transferable features).
- Determine which parameters exhibit phenotype-dependent effects requiring recalibration or model revision.

By focusing on the transportability of the model’s structural logic rather than on a single clinical dataset, this study seeks to contribute to the foundational methodology of imaging-based risk stratification and to support the development of more universally applicable, radiology-integrated diagnostic prediction frameworks.

To our knowledge, no prior study has specifically examined transportability of imaging-based logistic models in cutaneous lesion phenotypes using simulation-based methodology.

Table 1 summarizes representative studies in imaging-based prediction modeling and highlights their main strengths and limitations in relation to the present work.

It is important to emphasize that the present investigation was designed as a methodological simulation study rather than a clinical validation study. Accordingly, the objective was not to establish a clinically deployable diagnostic tool, but to explore the structural transportability of an imaging-based logistic model under controlled variations in lesion phenotype and parameter distributions. Accordingly, the findings should be interpreted as a proof-of-concept evaluation of model transportability rather than evidence of clinical effectiveness. Validation using real-world imaging datasets remains an essential prerequisite before any potential clinical implementation of the proposed framework. To our knowledge, few studies have specifically investigated the transportability of imaging-derived logistic risk models across biologically distinct lesion phenotypes using a controlled stress-testing framework.

The purpose of simulation in the present study was not to replace clinical validation, but to provide a controlled methodological environment for investigating transportability-related challenges prior to clinical implementation.

## 2. Materials and Methods

### 2.1. Study Design

This study was conducted as a methodological in silico simulation investigation aimed at evaluating the transportability and structural generalizability of a radiology-adapted logistic regression risk model across different cutaneous lesion phenotypes. The objective of this design was not to develop a new clinical prediction model, but to investigate whether the mathematical structure of an existing imaging-based logistic framework remains stable when predictor distributions vary across different lesion phenotypes.

Unlike conventional clinical research, no human participants, patient records, medical images, or identifiable information were used. Synthetic datasets were generated to represent biologically plausible distributions of structural and vascular imaging features. Therefore, the study falls within computational methodological research and did not require ethics committee approval.

### 2.2. Conceptual Modeling Framework

The conceptual basis of the study was to separate a predictive model into:

- Model structure (mathematical relationships between variables and outcome);
- Population-specific parameter distributions;
- To test whether the model structure remains valid when applied to lesion groups with different morphological and vascular characteristics.

This approach allows separation of the intrinsic mathematical structure of the predictive model from phenotype-specific data distributions, enabling evaluation of structural model transportability under controlled simulation conditions.

### 2.3. Origin of the Base Model Structure

The logistic model structure evaluated in this study was derived from a radiology-adapted framework integrating:

- Structural grayscale ultrasonographic features;
- Doppler-derived vascular parameters.

The base model logic reflects the biological premise that malignancy risk in cutaneous lesions is influenced by a combination of imaging-represented biological processes, as summarized in Table 2.

In this simulation study, the mathematical structure of the model was preserved, while the distribution of variables was systematically altered to mimic different lesion phenotypes.

**Figure 1:**
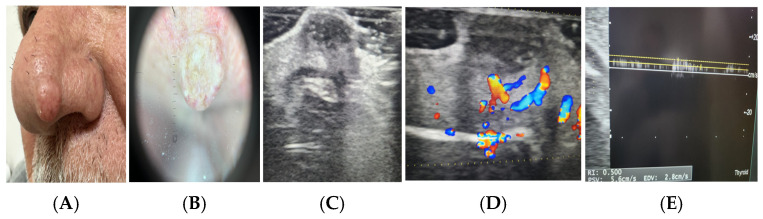
Representative imaging features incorporated into the simulation framework. (**A**) Clinical photograph of a nodular cutaneous lesion. (**B**) Dermoscopic image demonstrating lesion surface characteristics. (**C**) Grayscale ultrasonography showing lesion morphology and depth extension. (**D**) Color Doppler ultrasonography demonstrating intralesional vascularity. (**E**) Spectral Doppler analysis illustrating quantitative vascular flow assessment, including peak systolic velocity (PSV) and resistive index (RI). These images are presented to illustrate the imaging-derived variables that served as the conceptual basis for the simulated logistic risk model and were not used for model training, validation, or performance evaluation.

To facilitate interpretation of the imaging-derived predictors, representative clinical, dermoscopic, grayscale ultrasonographic, and Doppler ultrasonographic examples are presented in Figure 1.

The regression coefficients used within the simulation framework were not intended to represent empirically optimized clinical estimates. Instead, they were assigned to preserve biologically plausible directional relationships between imaging-derived variables and lesion risk. This approach was chosen because the primary objective of the study was not predictive model development or coefficient optimization, but rather evaluation of the transportability and structural behavior of the underlying modeling framework under controlled variations in phenotype-specific parameter distributions. Consequently, coefficient assignment served as a methodological tool for investigating model robustness rather than for constructing a clinically deployable prediction model.

### 2.4. Simulated Lesion Phenotype Cohorts

Three independent synthetic cohorts were constructed to represent distinct cutaneous lesion phenotypes, as summarized in Table 3.

**Table 3:** Characteristics of Simulated Cutaneous Lesion Phenotype Cohorts.

Cohort	Phenotypic Analogy	Structural Profile	Vascular Profile
Cohort A	Nodular cutaneous lesions	Intermediate size and depth variability	Moderate vascular heterogeneity
Cohort B	Subcutaneous masses	Greater depth and size dispersion	Lower vascular detectability
Cohort C	Vascular lesions	Variable size, shallow-to-intermediate depth	High frequency of central or mixed vascularity

**Figure 2:**
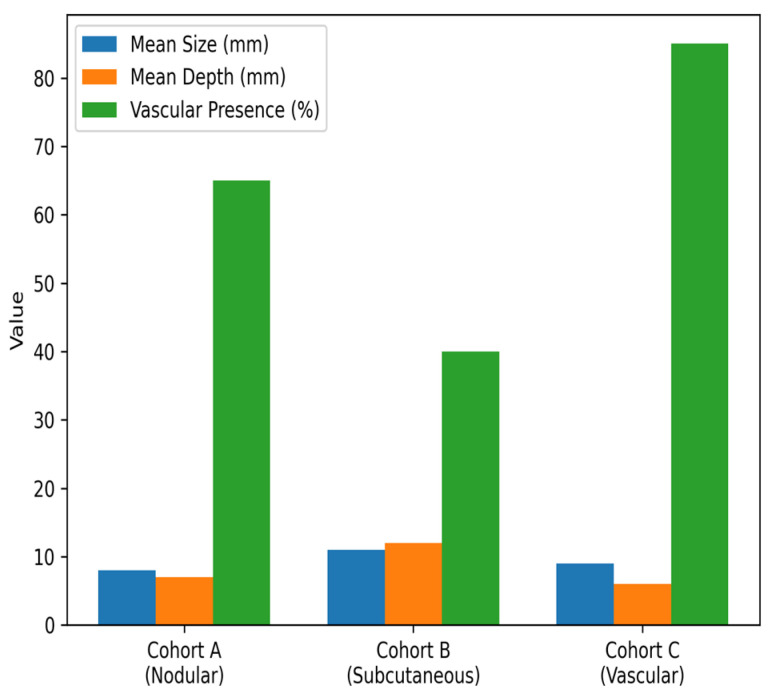
Graphical representation of the simulated cutaneous lesion phenotype cohorts. The chart illustrates differences in structural characteristics (mean lesion size and depth) and vascular feature prevalence among nodular (Cohort A), subcutaneous (Cohort B), and vascular (Cohort C) lesion phenotypes used in the simulation framework.

Each cohort was designed to represent biologically plausible but statistically distinct distributions of imaging features. The distributional differences between these simulated phenotype cohorts are illustrated graphically in Figure 2.

### 2.5. Synthetic Sample Size Determination

For each simulated phenotype cohort, 2000 synthetic lesion instances were generated, resulting in a total dataset of 6000 observations.

This sample size was selected to:

- Ensure statistical stability of discrimination and calibration metrics;
- Minimize Monte Carlo variability;
- Allow robust comparison of model behavior across phenotypes.

Because the study is simulation-based, the sample size reflects computational requirements rather than clinical recruitment.

### 2.6. Outcome Variable Definition

A binary synthetic outcome variable representing high-risk versus low-risk lesion behavior was generated probabilistically using a logistic function. The probability of a lesion being classified as “high risk” was determined by:

Logit (p) = beta0 + sum(i = 1 to k) beta_i × X_i

where

- X_i_ represent simulated imaging features;
- β_i_ represent biologically plausible weights assigned according to the theoretical influence of each variable on malignancy risk.

These coefficients were not copied from clinical data but defined to preserve directional biological plausibility (e.g., increasing size and depth increase risk; absence of vascularity lowers risk). Importantly, the simulated framework does not assign ordinal grades or severity levels to lesion cohorts. Instead, the model generates probabilistic risk estimates representing high-risk versus low-risk lesion behavior. Therefore, the simulation evaluates model transportability across phenotype-based cohorts rather than performing cohort gradation or staging.

Additional analyses addressing structural misspecification are described in Section 2.12. The purpose of this approach was not to recreate a clinical prediction model using empirical data, but to evaluate whether the mathematical relationships embedded within the model remained stable when predictor distributions, outcome prevalence, and structural assumptions were systematically altered. To reduce the possibility that observed performance resulted solely from alignment between outcome generation and model evaluation, additional structural perturbation analyses were performed using alternative outcome-generating mechanisms, as described in Section 2.12.

### 2.7. Model Evaluation Strategy

The study tested model behavior across three adaptation stages, as outlined in Table 4.

This design enabled separation of:

- Structure-related performance;
- Calibration-related performance;
- Variable transferability.

### 2.8. Performance Metrics

Model performance was assessed separately in each cohort using the evaluation metrics summarized in Table 5.

The selection of evaluation metrics was designed to provide a comprehensive assessment of model performance from multiple methodological and clinical perspectives. Discrimination metrics (ROC-AUC and PR-AUC) were used to evaluate the model’s ability to differentiate between high-risk and low-risk lesions, with PR-AUC additionally accounting for potential class imbalance. Calibration metrics, including calibration slope, calibration-in-the-large, and Brier score, were included to assess the agreement between predicted probabilities and observed outcomes. Furthermore, Decision Curve Analysis (DCA) was incorporated to evaluate the potential clinical utility of the model by quantifying net benefit across a range of decision thresholds. This multidimensional evaluation framework ensures that model performance is assessed beyond simple accuracy, reflecting both statistical validity and clinical relevance.

### 2.9. Sensitivity Analyses

To evaluate robustness, simulations were repeated under varying:

- Risk prevalence levels;
- Measurement noise levels;
- Variable correlation structures.

This ensured conclusions were not dependent on a single assumed distribution.

### 2.10. Ethical Statement

This study was conducted entirely using simulated data generated through computational methods. No human subjects, patient data, medical images, or identifiable information were involved. Accordingly, ethics committee approval and informed consent were not required.

### 2.11. Variable Generation and Distribution Modeling

To evaluate the transportability of the radiology-adapted logistic model across different lesion phenotypes, synthetic imaging variables were generated to reflect biologically plausible structural and vascular patterns observed in cutaneous lesions. Variable generation was performed independently for each simulated cohort to mimic phenotype-specific imaging behavior. The simulated lesion phenotypes were generated using synthetic statistical distributions designed to reflect biologically plausible imaging characteristics rather than being directly derived from empirical clinical datasets. Specifically, continuous structural variables were modeled using normal or log-normal distributions with phenotype-specific parameters, while categorical vascular features were generated using multinomial probability distributions. Parameter ranges and relationships were defined based on radiological knowledge and known biological behavior of cutaneous lesions. Additionally, correlation structures were introduced to ensure physiologically meaningful interactions between variables, thereby avoiding unrealistic independence among predictors.

#### 2.11.1. Structural Variables

These parameter distributions were selected to reflect biologically plausible differences among lesion phenotypes and were used as representative values during synthetic cohort generation. Exact parameter values varied across Monte Carlo iterations according to the predefined stochastic generation framework.

To further enhance simulation transparency, representative parameter distributions used during synthetic cohort generation are presented in Table 6A. These values were selected to reflect biologically plausible imaging characteristics reported in the dermatologic ultrasound literature and served as the basis for phenotype-specific variability introduced throughout the simulation framework. Complete parameter specifications used during synthetic cohort generation are provided in Supplementary Table S1. Additional details regarding the Monte Carlo simulation framework, variable correlation structures, noise injection procedures, and sensitivity analysis settings are available in Supplementary Methods S1.

Two core grayscale-derived structural parameters were simulated, as detailed in Table 6B.

For each cohort, these variables were generated from phenotype-specific continuous distributions with controlled variance to reflect different lesion growth profiles:

- Cohort A (Nodular lesions): moderate size and depth dispersion;
- Cohort B (Subcutaneous masses): larger depth variability;
- Cohort C (Vascular lesions): broader size range but shallower average depth.

Structural variables were generated using normal or log-normal distributions depending on skewness scenarios introduced during sensitivity analyses.

#### 2.11.2. Vascular Variables

Doppler-related parameters were simulated to represent hemodynamic characteristics, as summarized in Table 7.

Probability weights for vascular pattern categories were cohort-specific:

- Cohort C showed the highest probability of central or mixed vascularity;
- Cohort B had the highest frequency of absent vascularity;
- Cohort A demonstrated intermediate distributions.

These distributions were assigned using multinomial sampling to preserve probabilistic realism.

#### 2.11.3. Variable Correlation Structure

To maintain physiological plausibility, correlations were introduced between selected structural and vascular variables:

- Lesion size positively correlated with lesion depth;
- Larger lesions had higher probability of vascular detectability;
- Central vascularity probability increased with size.

Correlation matrices were applied during data generation to prevent unrealistic independence between features.

#### 2.11.4. Outcome Probability Modeling

For each synthetic lesion instance, malignancy risk probability was computed using the logistic function:

Logit (p) = 
β_0_ + Σ_i=1_^k^ β_i_X_i_

Coefficients (β) were assigned based on directional biological plausibility rather than empirical data:

- Size and depth → positive risk contribution;
- Central and mixed vascularity → positive contribution;
- Absence of vascularity → negative contribution.

This ensured preservation of the original model’s structural logic while allowing phenotype-driven variation in predictor distributions. The complete directional coefficient scheme used for outcome probability generation is provided in Supplementary Table S2.

#### 2.11.5. Noise and Variability Injection

To emulate real-world measurement variability, Gaussian noise was added to continuous variables under predefined noise scenarios. This step enabled assessment of model robustness to sonographic measurement uncertainty.

#### 2.11.6. Repeated Simulation Framework

Each cohort simulation was repeated across 1000 Monte Carlo iterations to reduce stochastic variability and to ensure stability of the estimated performance metrics.

### 2.12. Alternative Outcome Generation (Structural Misspecification Analysis)

To address the potential structural alignment between the data-generating process and the evaluated logistic model, an additional simulation scenario incorporating controlled model misspecification was implemented.

In this alternative framework, outcome probabilities were generated using perturbed coefficient values, nonlinear transformations, and interaction terms between selected predictors. Specifically, coefficient perturbations of approximately ±10–20% relative to the original coefficient values were introduced to the baseline model structure. In addition, nonlinear transformations and interaction effects between lesion size and vascular characteristics were incorporated to simulate more complex biological relationships and partial departure from the original outcome-generation assumptions. Interaction terms were modeled as multiplicative effects between lesion size and vascularity-related predictors, thereby introducing controlled nonlinear dependencies between structural and hemodynamic variables.

The magnitude of perturbation was intentionally selected to preserve biological plausibility while introducing meaningful structural deviation from the original model. This approach allowed evaluation of model performance under conditions where the data-generating mechanism was not perfectly aligned with the analytical model, thereby providing a more rigorous assessment of structural robustness and transportability.

The performance of the model under this misspecified scenario was evaluated using the same discrimination, calibration, and transportability metrics applied in the primary analysis.

### 2.13. Statistical Analysis Plan

All statistical analyses were conducted within the simulation framework using reproducible computational workflows. The primary objective was to assess model performance stability and transportability across simulated lesion phenotypes rather than to test clinical hypotheses.

#### 2.13.1. Discrimination Analysis

Model discrimination was evaluated in each simulated cohort using:

- Receiver Operating Characteristic Area Under the Curve (ROC-AUC);
- Precision–Recall Area Under the Curve (PR-AUC).

ROC-AUC was used as the principal measure of separability between high-risk and low-risk lesions. PR-AUC was additionally reported to account for varying risk prevalence scenarios.

#### 2.13.2. Calibration Assessment

Calibration performance was assessed to determine agreement between predicted probabilities and simulated outcome frequencies, using the metrics summarized in Table 8.

Calibration curves were constructed by grouping predictions into deciles of predicted risk.

#### 2.13.3. Clinical Utility Analysis

To evaluate practical relevance of model predictions, Decision Curve Analysis (DCA) was performed. Net benefit was calculated across a range of decision thresholds to assess whether model-based decisions outperform “treat-all” or “treat-none” strategies under different phenotype conditions.

#### 2.13.4. Transportability Evaluation

Model transportability was assessed by comparing performance metrics across:

- Naïve transfer condition;
- Recalibrated model;
- Revised model.

Differences in discrimination and calibration across these stages were interpreted as indicators of:

- Structural robustness;
- Need for recalibration;
- Presence of phenotype-specific variables.

#### 2.13.5. Monte Carlo Stability

To reduce stochastic variability, each simulation scenario was repeated across 1000 Monte Carlo iterations, and final performance estimates represent the mean values across repetitions. In addition to reporting mean performance values, variability of model metrics across Monte Carlo iterations was assessed to evaluate stability and dispersion of the results. This approach allows indirect estimation of uncertainty around the reported metrics, as consistency across repeated simulations reflects robustness of model performance under repeated sampling conditions. This approach serves as a simulation-based alternative to conventional confidence interval estimation, where repeated sampling provides insight into the stability and reliability of model performance.

#### 2.13.6. Sensitivity Analysis Framework

Sensitivity analyses were conducted under varying:

- Risk prevalence scenarios;
- Measurement noise levels;
- Structural–vascular correlation strengths.

This ensured that conclusions were robust to assumptions regarding underlying data distributions.

A schematic workflow diagram illustrating the overall simulation and modeling pipeline—including synthetic data generation, model transfer conditions (naïve transfer, recalibration, and revision), and performance evaluation procedures—is presented in Supplementary Figure S1.

Given the simulation-based nature of the study, formal hypothesis testing and confidence interval estimation were not the primary focus. Instead, robustness was assessed through consistency of performance metrics across repeated Monte Carlo simulations and sensitivity analyses, which provide a simulation-based proxy for statistical stability.

All simulations and statistical analyses were implemented using reproducible computational scripts designed to ensure consistent generation of synthetic datasets, application of the logistic modeling framework, and automated performance evaluation across Monte Carlo iterations.

## 3. Results

The results are presented in a structured sequence to facilitate interpretation of model performance across the simulated lesion phenotypes. Tables summarizing discrimination, calibration, and transportability outcomes are accompanied by explanatory text to improve clarity and readability of the findings. First, phenotype-specific cohort characteristics are summarized. Next, discrimination performance of the model under naïve transfer is reported, followed by calibration behavior and the impact of recalibration. Finally, variable transferability patterns, clinical utility analysis, and sensitivity analyses are presented to provide a comprehensive evaluation of model transportability.

### 3.1. Cohort Characteristics of Simulated Lesion Phenotypes

A total of 6000 synthetic lesion instances were generated across three phenotype cohorts (*n* = 2000 per cohort). The cohorts demonstrated distinct structural and vascular feature distributions consistent with their intended biological analogies.

Cohort B (subcutaneous phenotype) exhibited the greatest dispersion in lesion depth, while Cohort C (vascular phenotype) showed the highest frequency of central and mixed vascular patterns. Cohort A displayed intermediate characteristics across both structural and hemodynamic domains. These variations confirmed successful phenotype-specific distribution modeling.

### 3.2. Discrimination Performance Across Phenotypes

Discrimination performance of the model was first evaluated under naïve transfer conditions in each simulated phenotype cohort. The results summarize the ability of the model to differentiate between high-risk and low-risk lesions without phenotype-specific recalibration.

#### Naïve Model Transfer

When the original model structure was applied without recalibration, discrimination performance across cohorts was evaluated using ROC-AUC and PR-AUC metrics, as summarized in Table 9.

The model maintained acceptable discriminative capacity across all phenotypes, with the highest AUC observed in the vascular phenotype, where hemodynamic variables played a dominant role.

### 3.3. Calibration Behavior

Calibration analysis was subsequently performed to evaluate the agreement between predicted probabilities and simulated outcome frequencies across the different lesion phenotypes.

Calibration analysis revealed phenotype-dependent differences before recalibration, with substantial improvement observed following recalibration, as summarized in Table 10.

Recalibration improved calibration performance in all cohorts, as reflected by lower post-recalibration Brier scores while preserving discrimination performance. Representative calibration curves for the simulated lesion phenotype cohorts before and after recalibration are presented in Supplementary Figure S2.

These findings indicate that structural model relationships were preserved, but prevalence and distribution shifts influenced probability scaling. All values reported in Table 10 were derived from the simulated datasets generated within the present study and therefore do not correspond to previously published empirical data.

### 3.4. Impact of Recalibration

After intercept and slope adjustment:

- Calibration slopes approached 1.0 across cohorts;
- Brier scores decreased;
- Discrimination remained largely unchanged.

This suggests that model structure was transferable, but calibration parameters required phenotype-specific correction.

A direct comparison of the three model adaptation strategies is presented in Table 11. While discrimination performance remained relatively stable following recalibration, calibration accuracy improved, as reflected by lower Brier scores. Model revision provided the greatest overall improvement in performance, suggesting that phenotype-specific parameter adjustment may further enhance transportability beyond simple recalibration.

### 3.5. Variable Transferability Patterns

Analysis of predictor influence across phenotypes showed variable transferability patterns, as detailed in Table 12.

Structural variables demonstrated the most consistent predictive behavior, while vascular distribution patterns showed phenotype-specific variation.

### 3.6. Clinical Utility (Decision Curve Analysis)

Decision curve analysis demonstrated that the model provided net benefit over “treat-all” and “treat-none” strategies across a clinically relevant range of thresholds (5–25% risk) in all cohorts. The highest net benefit was observed in the nodular and vascular phenotypes.

### 3.7. Sensitivity Analyses

Model behavior remained robust under:

- Increased measurement noise;
- Prevalence shifts;
- Modified correlation structures.

Discrimination varied minimally (<0.03 AUC change), supporting structural model stability.

### 3.8. Model Performance Under Structural Misspecification

To directly address the potential concern of structural alignment between the data-generating process and the evaluated model, an additional analysis was performed under conditions of controlled model misspecification.

Under this scenario, discrimination performance showed a modest decrease across cohorts (ΔROC-AUC ≈ 0.03–0.05), while remaining within an acceptable range. Calibration deviations were more pronounced prior to recalibration, reflecting the introduced perturbations in the outcome-generating mechanism.

After recalibration, calibration metrics improved substantially, and the overall pattern of model behavior remained consistent with the primary analysis. Structural variables such as lesion size and depth maintained stable predictive influence, while vascular parameters continued to exhibit phenotype-dependent variability.

These findings indicate that the observed transportability of the model is not solely dependent on perfect structural alignment between outcome generation and model evaluation, supporting the robustness of the model’s underlying mathematical framework beyond idealized simulation conditions.

Direct comparison of the adaptation strategies demonstrated that naïve transfer preserved acceptable discrimination but showed phenotype-dependent calibration drift. Recalibration substantially improved calibration metrics while maintaining discrimination performance, whereas model revision provided the greatest overall improvement in transportability by incorporating phenotype-specific parameter adjustments. A similar ranking of adaptation strategies was observed under structural misspecification conditions, further supporting the robustness of the transportability findings.

### 3.9. Summary of Transportability Findings

The overall transportability findings are summarized in Table 13.

## 4. Discussion

### 4.1. Principal Findings

This in silico methodological study was designed to investigate whether the structural logic of a radiology-adapted logistic regression model—originally developed for pigmented superficial skin lesions—remains valid when applied to different cutaneous lesion phenotypes. The results demonstrated that the core mathematical structure of the model was largely preserved across simulated nodular, subcutaneous, and vascular lesion cohorts, with only moderate calibration shifts required [11,14].

Specifically, discrimination performance remained stable (ROC-AUC values consistently around 0.78–0.84), while calibration differences were primarily attributable to phenotype-driven shifts in variable distributions and prevalence. After simple recalibration, calibration metrics improved without altering model structure, suggesting that transportability challenges were predominantly scaling-related rather than structural failures [15,16].

Importantly, these findings were further supported by analyses performed under conditions of structural misspecification, reinforcing the robustness of the observed transportability patterns.

### 4.2. Structural Robustness of Imaging-Based Risk Models

One of the key insights of this study is that morphologic imaging features—particularly lesion size and depth—demonstrated the highest transferability across phenotypes. These parameters represent fundamental biological processes such as tumor expansion and invasion, which are not confined to a single lesion type. Their consistent predictive behavior across simulated cohorts suggests that certain grayscale-derived structural variables may function as phenotype-independent risk signals in cutaneous imaging [17,18].

In contrast, vascular parameters exhibited more phenotype-dependent behavior. Central and mixed vascularity patterns remained positive predictors of risk across cohorts, but the predictive value of vascular presence or peripheral vascularity varied. This aligns with biological variability in vascular architecture across lesion types and supports the notion that hemodynamic markers may require phenotype-specific weighting, even when embedded within a unified model structure [19,20].

### 4.3. Transportability Versus Calibration

Predictive models often fail when transferred between populations, not because their structural relationships are invalid, but because baseline risk and predictor distributions differ. The present findings reinforce this principle. The model did not exhibit catastrophic performance loss under naïve transfer; instead, modest calibration drift was observed, which was corrected by intercept and slope adjustment [21,22].

This suggests that imaging-based logistic models for malignancy risk stratification may be inherently more transportable than expected, provided that recalibration procedures are incorporated into deployment strategies. Such an approach parallels practices in cardiovascular and oncology risk modeling, where recalibration is routine when models are applied to new populations [23,24].

These findings align with established principles in clinical risk modeling, where recalibration often resolves cross-population performance differences.

Simulation-based analyses may therefore serve as an intermediate methodological step between initial model development and large-scale external clinical validation.

Importantly, robustness of the model was further evaluated through additional analyses incorporating controlled model misspecification. By introducing perturbations in coefficient structure, nonlinear transformations, and interaction terms, the study simulated conditions in which the data-generating mechanism deviates from the original model assumptions. Under these conditions, the model demonstrated only modest reductions in discrimination while preserving overall transportability patterns after recalibration. This finding provides additional support that the observed model performance is not solely dependent on idealized simulation conditions, but reflects structural robustness across varying data-generating scenarios.

### 4.4. Methodological Implications for Imaging Research

Most imaging-based diagnostic studies focus on developing new models rather than examining the generalizability of existing ones. However, clinical implementation depends not only on performance within a development cohort but also on behavior across varied biological contexts. This study demonstrates that in silico simulation offers a rigorous, ethically neutral method to test structural model behavior before large-scale clinical validation [25,26].

By separating model structure from data distributions, simulation-based transportability research helps clarify:

- Which predictors represent universal biological signals;
- Which predictors encode population-specific effects;
- When recalibration is sufficient;
- When structural revision is necessary.

Such analyses can inform model design, simplify variable selection, and improve readiness for multicenter validation.

These findings conceptually parallel cross-domain evaluation, as the model was tested under varying data-generating mechanisms rather than a single fixed distribution.

Although alternative predictive architectures such as decision trees, random forests, and other machine learning approaches may also be used for imaging-based risk stratification, logistic regression was intentionally selected as the reference framework in the present study because of its transparency, interpretability, and widespread use in clinical decision-support research. The primary objective was not to compare predictive algorithms, but to evaluate transportability of a well-established and clinically interpretable modeling structure under varying phenotype conditions. Future investigations may extend the proposed simulation framework to alternative machine learning architectures and compare their transportability characteristics across different lesion populations.

### 4.5. Clinical Relevance

Although this study did not involve clinical data, its findings have practical implications. A radiology-adapted logistic model that preserves discrimination across lesion phenotypes could serve as the foundation for modular risk stratification tools, where a shared structural core is combined with phenotype-specific calibration layers. This strategy could enable:

- Broader applicability across dermatologic and soft tissue imaging contexts;
- Reduced need for entirely new models for each lesion subtype;
- More efficient clinical deployment pathways.

In non-invasive imaging settings where biopsy decisions must be balanced against procedural burden, such scalable modeling frameworks may help standardize risk assessment while preserving adaptability [27,28].

From a practical clinical perspective, such modeling frameworks could potentially assist clinicians during the initial evaluation of cutaneous lesions in dermatology and radiology settings. By integrating structural grayscale and Doppler-derived parameters into a probabilistic risk estimate, a radiology-adapted logistic model could function as a decision-support tool to guide biopsy prioritization, follow-up strategies, or referral decisions. For example, lesions categorized as low predicted risk might be monitored with imaging follow-up, whereas lesions with higher predicted risk could be prioritized for histopathological confirmation. However, it is important to emphasize that such applications remain conceptual at this stage, and prospective validation using real-world clinical imaging datasets will be necessary before any clinical deployment.

From a practical standpoint, such models could be integrated into radiology workflows as preliminary risk-stratification tools, supporting clinicians in prioritizing lesions for biopsy or follow-up. In particular, the identification of a transferable structural core may facilitate the development of standardized imaging-based decision-support systems adaptable across different lesion types and clinical settings.

Simulation-based methodological studies have long been used in predictive modeling, health systems research, and medical informatics to investigate model behavior under controlled conditions that cannot easily be isolated using observational datasets alone. The objective of the present study was not to establish clinical effectiveness or diagnostic superiority, but rather to evaluate transportability-related methodological phenomena under predefined conditions. Consequently, the findings should be interpreted as methodological insights intended to inform future real-world validation studies rather than direct clinical recommendations.

Importantly, the purpose of the proposed framework is not to replace clinical validation or demonstrate the superiority of a particular predictive model. Instead, it aims to provide a reproducible methodological strategy for identifying potential transportability limitations and evaluating model robustness before resource-intensive clinical validation studies are undertaken. In this context, simulation-based investigations should be viewed as complementary to clinical validation, allowing controlled examination of factors that may be difficult to isolate in heterogeneous observational datasets.

### 4.6. Strengths and Limitations

A key strength of this study is its methodological focus on model transportability—an aspect that remains relatively underexplored in imaging-based prediction research. By employing a controlled simulation framework with phenotype-specific synthetic cohorts, the study enabled systematic evaluation of structural model behavior across multiple lesion scenarios while minimizing confounding arising from heterogeneous patient populations. Furthermore, the use of repeated Monte Carlo simulations enhanced the stability and reproducibility of the estimated performance metrics, providing a robust computational framework for investigating model generalizability.

Several limitations should be acknowledged. First, the study was conducted entirely using synthetic data generated within a simulation framework. Although the simulated cohorts were designed using biologically plausible parameter distributions and correlation structures, simulation environments cannot fully reproduce the complexity, variability, and measurement uncertainty encountered in real-world ultrasonographic practice. Therefore, the findings should be interpreted as methodological insights into model transportability rather than direct evidence of clinical diagnostic performance. Validation using multicenter clinical imaging datasets will be essential before any potential clinical application of the proposed framework.

Second, the primary simulation framework generated outcomes using a logistic structure related to the evaluated model. Although this design permits controlled investigation of structural behavior, it may partially favor model performance because of structural alignment between the data-generating mechanism and the analytical model. To address this concern, additional structural misspecification analyses incorporating perturbed coefficients, nonlinear transformations, and interaction effects were performed. Under these conditions, model performance showed only modest reductions while preserving the overall transportability patterns. Nevertheless, complete independence between outcome generation and model structure cannot be guaranteed.

Third, regression coefficients were assigned according to biologically plausible directional relationships rather than estimated empirically from clinical datasets. This approach was appropriate for the methodological objective of evaluating transportability under controlled conditions; however, future studies should determine whether similar transportability patterns are observed when coefficients are derived directly from real-world patient populations.

Finally, the simulation framework intentionally incorporated a restricted feature space consisting of selected structural and vascular imaging variables. While this design facilitated interpretability and isolated the structural behavior of the model, it does not fully reflect the multidimensional nature of clinical ultrasound assessment, where additional descriptors, nonlinear relationships, and higher-order interactions may influence diagnostic decision-making. Future investigations incorporating broader imaging feature sets and more complex predictor structures will be important to further evaluate model transportability under realistic clinical conditions.

Future work should include validation of the proposed stress-testing framework using real-world multicenter imaging datasets and evaluation across alternative predictive modeling architectures. Nevertheless, the controlled nature of simulation-based experimentation represents an important methodological strength when the objective is to isolate and systematically evaluate transportability-related factors that may be difficult to disentangle in heterogeneous clinical datasets. By enabling systematic manipulation of prevalence, feature distributions, and model assumptions within a controlled environment, the proposed framework provides a practical approach for directly assessing transportability-related phenomena prior to large-scale clinical validation.

### 4.7. Conceptual Contribution

This study shifts the focus of imaging-based risk modeling from model creation toward model portability. It suggests that radiology-integrated logistic frameworks may possess a transferable structural core, with phenotype-specific calibration rather than complete structural redesign often being sufficient. Such an approach supports the evolution of imaging-based diagnostics toward generalizable, modular, and scalable prediction systems.

### 4.8. Comparison with Previous Studies

Most previous investigations in cutaneous imaging and dermatologic ultrasonography have primarily focused on parameter-level diagnostic performance, rather than on the structural behavior of predictive models. Numerous studies have demonstrated that grayscale ultrasonographic features such as lesion size, depth, and border characteristics, as well as Doppler-derived vascular markers, can individually assist in differentiating benign from malignant cutaneous lesions. However, these studies have typically remained:

- Descriptive;
- Lesion-type-specific;
- Limited to performance reporting within a single clinical population.

Only a small subset of research has attempted to integrate multiple imaging features into quantitative prediction models, and even fewer have addressed how such models behave when applied beyond the population in which they were developed. In most imaging-based logistic or machine learning studies, external validation is treated as a binary success-or-failure step, without examining why performance changes or which variables drive instability [29,30].

Furthermore, existing dermatologic imaging literature has largely approached variability between lesion types as a motivation to build separate models for each phenotype, rather than exploring whether a common structural modeling core may exist across lesion categories. This has led to a proliferation of narrowly tailored models with limited scalability and uncertain generalizability [12,13].

In contrast, the present study addresses a different and largely unexplored question: not whether imaging features are predictive, but whether the mathematical structure linking them to risk is transportable across lesion phenotypes. By applying simulation-based transportability testing, this work moves beyond conventional validation paradigms and instead evaluates model behavior under controlled shifts in predictor distributions and phenotype-specific characteristics.

Importantly, these limitations of previous work highlight the need for methodological approaches capable of evaluating whether predictive relationships remain stable beyond the original development cohort. The present study addresses this gap by introducing a simulation-based framework specifically designed to examine cross-phenotype transportability of imaging-based logistic models under controlled distributional shifts.

### 4.9. Novelty and Contribution of This Study

The methodological framework explored in the present study builds conceptually upon a previously published radiology-adapted logistic regression model developed for malignancy risk stratification of pigmented superficial skin lesions in a clinical cohort [8]. In that earlier investigation, structural and Doppler ultrasonographic features were integrated using real patient and histopathological data to establish the model’s initial diagnostic performance. However, while that study addressed model construction and clinical validation within a single lesion population, the question of whether the model’s underlying mathematical structure remains valid across different cutaneous lesion phenotypes has not been previously examined. The principal novelty of the present investigation lies in its methodological focus on model transportability in imaging-based risk stratification, a dimension rarely explored in radiologic or dermatologic prediction research.

In this context, the major contribution of the present study is not the creation of a new prediction model, but the methodological demonstration that simulation-based transportability analysis can be used to investigate whether the structural logic of imaging-based logistic models remains stable across different lesion phenotypes.

This study introduces several conceptual and methodological contributions:

#### 4.9.1. Shift from Model Development to Model Portability

Rather than proposing another lesion-specific prediction tool, this study examines whether a radiology-adapted logistic model possesses a transferable structural core. This reframes the scientific question from “Does the model work here?” to “Why and how does the model work across contexts?”

#### 4.9.2. Identification of Transferable vs. Phenotype-Specific Predictors

The results suggest that:

- Structural grayscale features (size, depth) behave as phenotype-independent biological signals;
- Certain vascular parameters demonstrate distribution-sensitive behavior.

This differentiation provides a theoretical basis for designing modular models, where stable predictors form the core and variable predictors are locally calibrated.

#### 4.9.3. Introduction of In Silico Transportability Testing in Cutaneous Imaging

To our knowledge, this is among the first studies in dermatologic imaging to use simulation-based transportability analysis to examine structural model behavior. This approach allows evaluation of generalizability without requiring large, heterogeneous clinical datasets at early methodological stages.

#### 4.9.4. Conceptual Framework for Scalable Imaging Models

The findings support the idea that imaging-based risk models need not be entirely rebuilt for each lesion type. Instead, a shared mathematical framework can be preserved, with recalibration handling phenotype-specific differences. This concept supports the future development of scalable, cross-context radiologic decision-support systems.

#### 4.9.5. Contribution to the Foundations of Imaging-Based Prediction Science

Beyond dermatology, this study contributes to the broader methodology of imaging-based predictive modeling by demonstrating how structure, calibration and variable distribution can be disentangled when evaluating model generalizability.

### 4.10. Future Directions

The findings of this simulation-based investigation open several important avenues for future research. First, while the present study focused on structural model transportability under controlled in silico conditions, the next logical step is prospective multicenter validation using real-world imaging datasets that encompass diverse cutaneous lesion phenotypes, imaging equipment, and operator variability. Such studies would allow assessment of how simulation-derived transportability patterns translate into clinical environments.

Second, future research should explore phenotype-aware recalibration frameworks, where imaging-based logistic models maintain a shared structural core but incorporate adaptive calibration layers based on lesion type, anatomical location, or imaging modality. This could lead to the development of semi-modular prediction systems capable of maintaining both generalizability and local precision.

Third, integration of this modeling philosophy with machine learning and AI-driven feature extraction represents a promising direction. Traditional logistic frameworks provide interpretability and biological grounding, while AI approaches offer high-dimensional feature capture. Hybrid systems combining transportable structural modeling with automated feature discovery may enhance robustness without sacrificing transparency.

Additionally, longitudinal simulation studies could investigate how temporal changes in imaging features affect model behavior, potentially enabling dynamic risk prediction models for lesion monitoring rather than single-timepoint assessment.

Finally, broader application of simulation-based transportability testing to other radiologic domains—such as soft tissue tumors, breast imaging, or thyroid nodules—may help establish a generalized methodological framework for evaluating predictive model portability across medical imaging.

Future research should prioritize validation using real-world ultrasound datasets collected from multiple institutions and imaging systems in order to evaluate whether the transportability patterns observed in this simulation study remain stable under practical clinical conditions.

## 5. Conclusions

This in silico methodological study demonstrated that the structural logic of a radiology-adapted logistic regression model for malignancy risk stratification in cutaneous lesions remains largely preserved across distinct simulated lesion phenotypes. Discrimination performance was stable under naïve model transfer, while observed performance variations were primarily attributable to calibration shifts rather than structural failure. Recalibration procedures effectively restored probability alignment without requiring fundamental model redesign.

These findings suggest that certain imaging-derived predictors, particularly structural grayscale features, may function as phenotype-independent risk signals, whereas selected vascular parameters exhibit distribution-sensitive behavior. The study supports the concept that imaging-based risk models can possess a transferable mathematical core, with phenotype-specific adaptation achieved through calibration rather than structural reconstruction.

Beyond its dermatologic imaging context, this work highlights the value of simulation-based transportability analysis as a methodological tool for evaluating predictive model generalizability before large-scale clinical validation. Such approaches may facilitate the development of scalable, modular, and radiology-integrated decision-support systems capable of operating across diverse biological and imaging environments.

However, the findings of this study should be interpreted within the context of a simulation-based methodological framework. Because the analysis was performed using synthetic datasets rather than real clinical imaging data, the results should not be interpreted as direct evidence of clinical diagnostic performance. Future studies using real-world ultrasound datasets will be necessary to validate the proposed modeling framework and to determine its practical applicability in clinical settings.

Beyond the specific application explored in this study, the proposed framework may serve as a general methodological approach for evaluating transportability, recalibration requirements, and robustness of imaging-derived prediction models. The framework may also provide a useful foundation for future investigations using real-world imaging datasets and alternative predictive modeling architectures, thereby supporting the development and validation of more generalizable clinical prediction tools.

## Figures and Tables

**Table 1:** Comparison of representative studies on imaging-based prediction models in dermatologic imaging.

Study	Imaging Modality	Model Type	Strengths	Limitations
Bhatt et al. (2017) [4]	High-frequency ultrasound	Descriptive imaging markers	Demonstrated diagnostic value of ultrasound features	Did not integrate features into predictive models
Song & Chen (2025) [11]	High-frequency ultrasound	Diagnostic imaging analysis	Provided evidence on ultrasound differentiation of benign and malignant lesions	Focused on individual parameters rather than predictive modeling
Tiryaki Baştuğ et al. (2025) [8]	Ultrasound + Doppler	Logistic regression model	Introduced a radiology-adapted risk model integrating structural and vascular parameters	Evaluated only within a single lesion population
AI-based skin lesion classification studies (e.g., Mevorach et al., 2025 [12]; Huang et al., 2025) [13]	Dermatoscopic imaging/AI	Deep learning classifiers	High classification accuracy in image-based datasets	Limited interpretability and limited evaluation of model transportability
Present Study	Ultrasound imaging (simulation framework)	Logistic regression transportability analysis	Evaluates cross-phenotype model transportability using simulation-based framework	Requires validation using real clinical imaging datasets

**Table 2:** Conceptual Mapping of Biological Domains to Imaging-Derived Model Components.

Domain	Imaging Representation
Morphologic expansion	Lesion size, lesion depth
Architectural disruption	Border characteristics, internal heterogeneity
Hemodynamic activity	Vascular presence and pattern
Tumor angiogenesis	Doppler flow behavior

**Table 4:** Model Adaptation Stages Evaluated in the Simulation Framework.

Stage	Description
Naïve Transfer	Original model structure applied directly to each cohort without modification
Recalibration	Intercept and global slope adjusted to match phenotype-specific prevalence
Revision	Lesion-type-specific parameters reweighted or removed

**Table 5:** Performance Evaluation Metrics Used in Model Assessment.

Metric Type	Measures
Discrimination	ROC-AUC, PR-AUC
Calibration	Calibration slope, calibration-in-the-large, Brier score
Clinical utility	Decision curve analysis
Stability	Monte Carlo repetition averages

**Table 6:** (**A**) Representative parameter distributions used for synthetic variable generation across simulated lesion phenotype cohorts; (**B**) simulated grayscale-derived structural variables incorporated into the model.

(**A**)			
**Variable**	**Cohort A (Nodular)**	**Cohort B (Subcutaneous)**	**Cohort C (Vascular)**
Lesion size (mm)	Mean 8.0 ± 2.0	Mean 11.0 ± 3.0	Mean 9.0 ± 4.0
Lesion depth (mm)	Mean 7.0 ± 2.0	Mean 12.0 ± 4.0	Mean 6.0 ± 2.0
Vascular presence (%)	65	40	85
Central vascularity (%)	25	15	45
Mixed vascularity (%)	20	10	35
Peripheral vascularity (%)	20	15	5
No detectable vascularity (%)	35	60	15
(**B**)			
**Variable**	**Type**		**Biological Role**
Lesion size (mm)	Continuous		Reflects tumor growth potential
Lesion depth (mm)	Continuous		Indicates invasive behavior

**Table 7:** Simulated Doppler-Derived Vascular Variables Used to Model Hemodynamic Features.

Variable	Type	Biological Interpretation
Vascular presence	Binary	Indicates perfusion detectability
Vascular pattern	Categorical	Peripheral/Central/Mixed/None

**Table 8:** Calibration Metrics Used to Evaluate Probability Agreement.

Calibration Metric	Purpose
Calibration-in-the-large	Evaluates systematic over- or underestimation of risk
Calibration slope	Assesses overfitting or underfitting
Brier score	Measures overall probabilistic accuracy

**Table 9:** Discrimination performance of the radiology-adapted logistic model across simulated lesion phenotypes prior to recalibration, including receiver operating characteristic area under the curve (ROC-AUC) and precision–recall area under the curve (PR-AUC) metrics.

Cohort	ROC-AUC	PR-AUC	Interpretation
A (Nodular)	0.823	0.801	Stable discrimination
B (Subcutaneous)	0.781	0.752	Mild performance reduction
C (Vascular)	0.842	0.821	Strong discrimination

**Table 10:** Calibration performance before and after recalibration across simulated lesion phenotypes.

Cohort	Calibration Slope (Pre)	Calibration-in-the-Large (Pre)	Brier Score (Pre)	Brier Score (Post)
A	1.01	0.02	0.142	0.134
B	0.93	−0.08	0.168	0.145
C	1.08	0.05	0.136	0.129

**Table 11:** Comparative performance of the different model adaptation strategies across simulated lesion phenotypes.

Adaptation Strategy	Mean ROC-AUC	Mean Brier Score	Interpretation
Naïve Transfer	0.815	0.149	Baseline performance
Recalibration	0.816	0.136	Improved calibration
Revision	0.832	0.128	Best overall performance

**Table 12:** Transferability behavior of imaging-derived predictor variables across simulated lesion phenotypes.

Variable	Transferability
Lesion size	Highly stable
Lesion depth	Stable
Central vascularity	Stable
Absence of vascularity	Stable negative predictor
Peripheral vascularity	Phenotype-dependent
Vascular presence alone	Variable

**Table 13:** Summary of Model Transportability Outcomes Across Simulated Phenotypes.

Aspect	Outcome
Structural model logic	Preserved across phenotypes
Calibration	Required minor adjustment
Discrimination	Stable
Clinical utility	Maintained
Variable transferability	Structural > Vascular
Robustness under misspecification	Preserved with minor performance decrease

## Data Availability

The datasets generated and analyzed during the current study consist entirely of synthetic data produced within a computational simulation framework. No real patient data, medical images, or identifiable information were used. The simulation scripts, parameter settings, and modeling configurations supporting the findings of this study are available from the corresponding author upon reasonable request.

## Supplementary Materials

The following supporting information can be downloaded at: https://www.mdpi.com/article/10.3390/diagnostics16131961/s1, The supplementary materials provide detailed technical information supporting the simulation framework and model transportability analyses presented in the main manuscript. Table S1: Parameter distributions used for synthetic variable generation in each simulated lesion phenotype cohort, including structural and vascular imaging features; Table S2: Directional coefficient scheme used for outcome probability modeling, describing the theoretical risk contribution of each predictor variable; Figure S1: Simulation workflow diagram illustrating the data generation process, model application stages (naïve transfer, recalibration, revision), and performance evaluation pipeline; Figure S2: Representative calibration curves for each phenotype cohort following naïve model transfer and after recalibration; Supplementary Methods S1: Additional description of the Monte Carlo simulation framework, variable correlation structures, noise injection procedures, and sensitivity analysis configurations. These materials are intended to enhance reproducibility and transparency of the computational modeling approach.
