# Supplementary material for: A Simulation-Based Stress-Testing Framework for Evaluating the Transportability of Imaging-Derived Logistic Risk Models Across Cutaneous Lesion Phenotypes
Source: Diagnostics (Basel). 2026 Jun 24;16(13):1961. doi: 10.3390/diagnostics16131961 (PMC13359699; doi:10.3390/diagnostics16131961)
Supplement: Supplementary file 1 [file diagnostics-16-01961-s001.zip › diagnostics-4330905-supplementary.pdf]

Supplementary Methods

Monte Carlo simulations were performed using phenotype-specific parameter distributions. Continuous variables were generated from normal distributions, while vascular features were assigned using Bernoulli and multinomial sampling. Correlation matrices were applied to preserve physiological relationships between structural and vascular parameters. Gaussian noise was introduced to emulate measurement variability encountered in real-world ultrasonographic assessment. Each simulation scenario was repeated across 1,000 Monte Carlo iterations to obtain stable performance estimates.

These procedures ensured that the simulation framework evaluated model structural behavior rather than performance within a specific clinical dataset.

Parameter distributions were not derived from a specific patient cohort but were defined to reflect biologically plausible imaging behavior and directional relationships reported in prior literature and the conceptual logic of the original model framework. The aim was to test structural model behavior under controlled distributional variation rather than to reproduce any single clinical dataset.

Supplementary Table S1. Plausible Parameter Distributions Used for Synthetic Variable Generation

Variable	Cohort A (Nodular)	Cohort B (Subcutaneous)	Cohort C (Vascular)	Distribution Type
Lesion Size (mm)	Mean = 8, SD = 3	Mean = 11, SD = 4	Mean = 9, SD = 5	Normal
Lesion Depth (mm)	Mean = 7, SD = 3	Mean = 12, SD = 5	Mean = 6, SD = 2	Normal
Vascular Presence	65% Yes / 35% No	40% Yes / 60% No	85% Yes / 15% No	Bernoulli
Vascular Pattern — None	35%	60%	15%	Multinomial
Vascular Pattern — Peripheral	30%	25%	20%	Multinomial
Vascular Pattern — Central	20%	10%	35%	Multinomial
Vascular Pattern — Mixed	15%	5%	30%	Multinomial

Parameter distributions were defined to represent biologically plausible imaging characteristics across different cutaneous lesion phenotypes. Continuous variables (lesion size and lesion depth) were generated using normal distributions with cohort-specific means and standard deviations. Vascular presence was simulated using Bernoulli sampling, while vascular pattern categories were generated using multinomial probabilities. These distributions were selected to reflect realistic variability in ultrasonographic lesion characteristics while maintaining controlled conditions for evaluating model transportability.

Supplementary Table S2. Directional Coefficient Scheme Used in Outcome Modeling

Predictor	Direction of Effect	Biological Interpretation
Lesion Size	Positive	Larger lesions associated with higher malignancy risk
Lesion Depth	Positive	Greater depth reflects invasive potential
Central Vascularity	Positive	Suggests tumor angiogenesis
Mixed Vascularity	Positive	Indicates complex perfusion patterns
Peripheral Vascularity	Variable	May differ by phenotype
Absence of Vascularity	Negative	More typical of benign lesions

Coefficient directions were defined to preserve biological plausibility and the conceptual logic of the base model rather than to reproduce empirically estimated clinical coefficients. The coefficient scheme reflects directional relationships commonly reported in imaging-based risk modeling of cutaneous lesions.

Supplementary Figure S1. Simulation Workflow Diagram

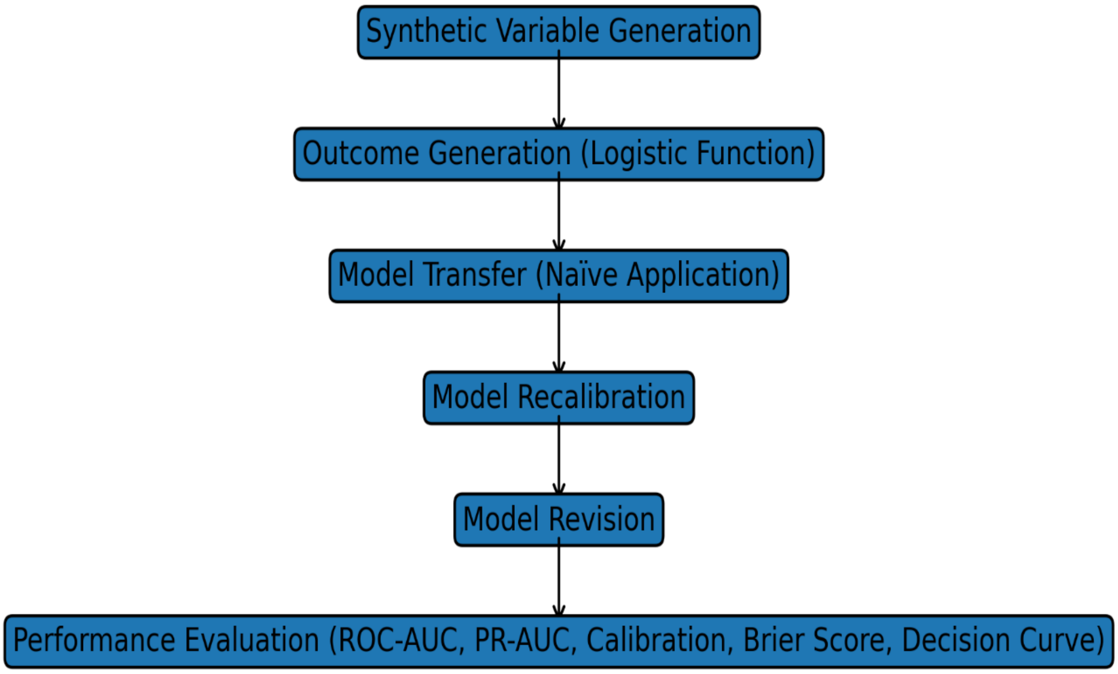

Workflow illustrating the generation of phenotype-specific synthetic datasets, application of the radiology-adapted logistic model under naïve transfer, recalibration, and revision stages, followed by evaluation of discrimination, calibration, and clinical utility across Monte Carlo iterations.

Supplementary Figure S2. Calibration Curves Across Cohorts

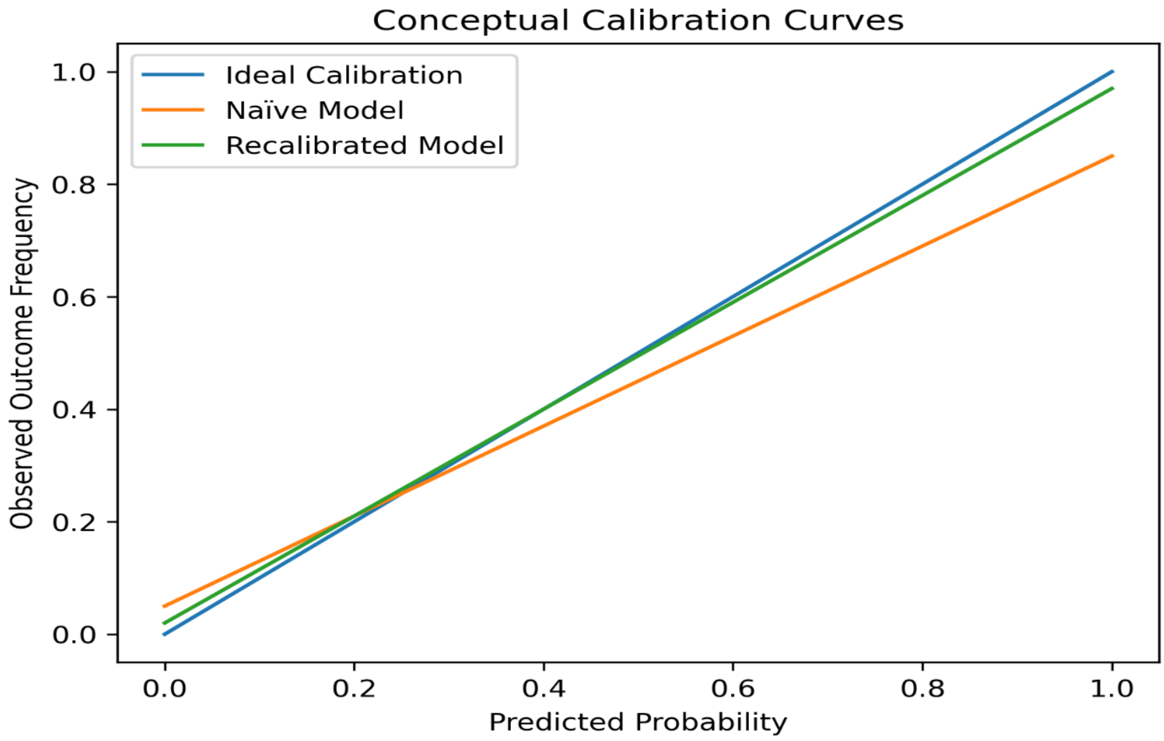

Representative calibration plots for each simulated phenotype cohort illustrating the agreement between predicted probabilities and observed outcomes under naïve model transfer and the improvement achieved after recalibration of intercept and slope parameters.
